# Periodontal health knowledge and awareness among subjects with fixed orthodontic appliance

**DOI:** 10.1590/2177-6709.23.5.40.e1-9.onl

**Published:** 2018

**Authors:** Elham S. Abu Alhaija, Eman M. Al-Saif, Dafi Q. Taani

**Affiliations:** 1 Jordan University of Science and Technology, Faculty of Dentistry, Department of Preventive Dentistry (Irbid, Jordan).

**Keywords:** Awareness, Attitude, Orthodontic, Periodontal health

## Abstract

**Objective:**

To report on periodontal health knowledge and awareness among orthodontic patients and to investigate the effect of age, attitude and duration of orthodontic treatment on periodontal health awareness among orthodontic patients.

**Methods:**

A total of 297 orthodontics patient (90 males, 207 females) with mean age of 17.7 ± 5.0 years (older then 18 years = 119, 18 years or younger = 178) were included in this study. Subjects were currently wearing upper and lower fixed orthodontic appliances for an mean period of 12.55 ± 10.86 months (less than or equal to 18 months = 231, more than 18 months = 66). Data was collected through a self-administered questionnaire (demographic characteristics, subjects’ awareness toward their periodontal health, periodontal knowledge among orthodontic patient and patients’ attitude toward orthodontic treatment regarding periodontal health) and clinical periodontal examination.

**Results:**

Periodontal knowledge was poor among orthodontic patients in relation to dental plaque. Only 24 subjects (8%) correctly answered knowledge-related questions. Adult orthodontic patients reported negative attitude toward fixed orthodontic treatment in respect to periodontal health (*p*<0.001). Duration of orthodontic treatment negatively affected subjects’ attitude toward fixed orthodontic treatment (*p*<0.01). The majority of subjects were in the high level of awareness group (64%). Orthodontic patients’ awareness toward their periodontal health during fixed orthodontic treatment was affected by attitude scores (*p*=0.005), number of teeth with gingival recession (*p*=0.041), Gingival Index (*p*=0.000), duration of treatment (*p*=0.047) and age (*p*=0.008).

**Conclusions:**

Periodontal health knowledge among orthodontic patients was poor. Orthodontic patients’ awareness of their periodontal health was moderate and was affected by age, attitude and duration of orthodontic treatment.

## Resumo

**Objetivo:**

relatar sobre o conhecimento e a conscientização quanto à saúde periodontal dos pacientes ortodônticos e investigar os efeitos da idade, atitude e duração do tratamento sobre a conscientização da saúde periodontal entre esses pacientes.

**Métodos:**

um total de 297 pacientes foi incluído nesse estudo (90 homens e 207 mulheres), com idade média de 17,7±5,0 anos (maiores de 18 anos = 119, iguais ou menores de 18 anos = 178). Os indivíduos estavam usando aparelhos ortodônticos fixos nas arcadas superior e inferior por um período médio de 12,55±10,86 meses (18 meses ou menos = 231, mais de 18 meses = 66). Os dados foram coletados por meio de um questionário autoaplicável (características demográficas, conscientização com relação à saúde periodontal, conhecimento periodontal entre os pacientes ortodônticos e a atitude dos pacientes com relação ao tratamento ortodôntico com relação à saúde periodontal) e exame clínico periodontal.

**Resultados:**

o conhecimento periodontal entre os pacientes ortodônticos foi pequeno com relação à placa bacteriana. Somente 24 indivíduos (8%) responderam corretamente às questões relacionadas ao conhecimento. Os pacientes ortodônticos adultos relataram uma atitude negativa quanto ao tratamento com aparelhos fixos, com relação à saúde periodontal (*p*<0,001). A duração do tratamento afetou negativamente a atitude dos indivíduos com relação ao tratamento com aparelhos fixos (*p*<0,01). A maioria dos indivíduos estava no nível mais alto do grupo conscientização (64%). A conscientização dos pacientes ortodônticos com relação à sua saúde periodontal durante o tratamento ortodôntico com aparelhos fixos foi afetada pelos escores da atitude (*p*=0,005), pelo número de dentes com retração gengival (*p*=0.041), Índice Gengival (*p*=0,000), duração do tratamento (*p*=0,047) e idade (*p*=0,008).

**Conclusões:**

o conhecimento sobre a saúde periodontal entre os pacientes foi pequeno. A conscientização dos pacientes quanto à sua saúde periodontal foi moderada e foi afetada pela idade, atitude e duração do tratamento ortodôntico.

## INTRODUCTION

Several studies showed a significant increase in the quantity of dental plaque and the occurrence of gingivitis in patients with fixed orthodontic appliances.^1^ For this reason, special efforts are required for adequate oral hygiene during fixed appliance treatment as its presence makes tooth cleaning more difficult.^2^

During fixed orthodontic appliance therapy, patient’s knowledge, motivation, cooperation and attitude toward treatment are key factors of oral hygiene maintenance.^3^ Poor maintenance of oral hygiene may be due to lack of knowledge or negligence by patients themselves.^4^ Various reports have shown that orthodontic patients’ knowledge on their gingival health was poor.^5^ Despite receiving appropriate instructions, many individuals fail to follow these instructions. Also, many of them lack knowledge on the maintenance of oral hygiene.

It has been documented that improvement of oral hygiene compliance and effectiveness during orthodontics can be achieved with professional instruction and monitoring.^6^ Before the beginning of orthodontic treatment, patients should be instructed about the importance of regular oral hygiene maintenance.^7^ It is necessary to demonstrate to patients the correct technique and frequency of tooth brushing. They need to learn about the right toothbrushes, interdental and orthodontic brushes, as well as the auxiliary devices for oral hygiene maintenance.^8^

Little is known about periodontal health knowledge and awareness among orthodontic patients. Therefore, the aims of this study were to report on the periodontal health knowledge and awareness among orthodontic patients, and to investigate the effect of age, attitude and duration of orthodontic treatment on periodontal health knowledge and awareness among orthodontic patients treated with fixed appliances.

### Null hypothesis 

A high periodontal health knowledge and awareness exist among subjects treated with fixed orthodontic appliances. Age, attitude and duration of treatment has no effect on periodontal health knowledge and awareness among orthodontic patients.

## MATERIAL AND METHODS

An ethical approval for the conduction of this study was obtained from the Institutional Review Board of Jordan University of Science and Technology (JUST, IRB #18/2011). A convenient sampling technique was adopted in the present study. A structured questionnaire was administered to 350 orthodontic patients recruited from orthodontic clinics at JUST. Currently treated orthodontic patients at the orthodontic clinics during the study period who agreed to participate in the study were included. The patients received both verbal and written information about the study. All subjects were of similar economic and social background.

A total of 297 orthodontics patient (90 males and 207 females) filled the questionnaire completely and were included in this study (Response rate was 85%). Age averaged 17.7±5.0 years (119 subjects were below 18 years, age range 15 to 17 years; and 178 subjects were 18 years or above, age range 18 to 29 years). Educational level in years was recorded, which ranged from 8 to 17 years. Fifty-nine percent of subjects were school children and the rest were university students.

Subjects were currently wearing upper and lower fixed orthodontic appliances for an average duration of 12.55±10.86 months (231 subjects wearing fixed orthodontic appliance for less than or equal to 18 months). Treatment duration ranged from 2 to 18 months, and 66 subjects were wearing fixed orthodontic appliances for more than 18 months (treatment duration ranged from 19 to 48 months). All subjects had healthy periodontium before the start of orthodontic treatment and were instructed for the need to properly clean the teeth after placing the appliance. Patients with cognitive disorders or chronic medical conditions, craniofacial anomalies such as cleft lip and palate and aggressive periodontitis were excluded. 

**Figure 1:**
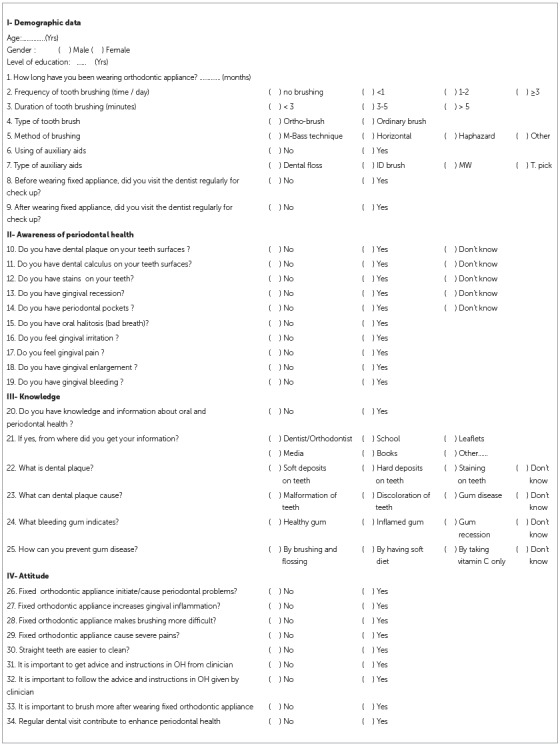
Periodontal health knowledge and awareness questionnaire answered by orthodontic patients in Northern Jordan.

A self-administrated structured questionnaire, which was applied in previous study^9^ was used as the instrument for data collection (Fig 1). The questionnaire was self-administered to the participants in the waiting areas of orthodontic clinics. It contained a series of questions in relation to demographic characteristics of the subjects age, gender, duration of fixed orthodontic treatment, the patient’s current oral health behavior (frequency and duration of tooth brushing, auxiliary aids and dental visits). 

Ten questions related to subject’s awareness toward periodontal health were scored as 0 if the answer was *"I don’t know"* and as 1 if the answer was *"yes"* or *"no"*. Subsequently, subjects were allocated into a high level of awareness (average score 8-10), a moderate level of awareness (5-7), a low level of awareness (average score 1-4) and no awareness (score 0) groups. 

Questions related to periodontal knowledge included what is dental plaque, what can dental plaque cause, what does bleeding gum indicate and how to prevent gum disease. Answers were given in the form of multiple choice, with only one correct answer. Questions related to subject’s knowledge of periodontal health were scored as 0 if the answer was correct and as 1 if the answer was incorrect. 

Questions related to subject’s attitude toward orthodontic treatment and periodontal health were scored as 0 if the answer was negative and as 1 if the answer was positive. Subsequently, subjects were allocated into positive attitude (average score 6-9) and negative attitude (average score 0-5) groups. 

Clinical examination was performed at orthodontic clinics at Jordan University of Science and Technology Dental Teaching Center. Mouth mirrors and periodontal probe (Williams’s style) markings at 1, 2, 3, 5, 8, 9, and 10 mm were employed for the inspection of the teeth surfaces. Clinical examination included gingival index^11^ (GI), plaque index^10^ (PI), probing pocket depth (PPD) and number of teeth with gingival recession. PI and GI were evaluated for each subject. Six representative teeth (Ramfjord teeth) were chosen for this purpose: maxillary right lateral incisor, maxillary right first premolar, maxillary right first molar, mandibular left lateral incisor, mandibular left first premolar and mandibular left first molar.^12^ If any of these pre-selected teeth was missing, the adjacent tooth was scored. The average score of each tooth was calculated by dividing the total score of each tooth surface by the number of surfaces examined. 

PPD was measured from the crest of the gingival margin to the base of periodontal pocket. Measurements were made in six sites (distobuccal, midbuccal, mesiobuccal, distolingual, midlingual, mesiolingual) of each tooth. The probe with accurate millimeter scale was inserted into the deepest part of gingival sulcus until resistance appeared. PPD was measured to the nearest millimeter on the scale.

### Reliability

Clinical examination was preceded by a period of training and calibration of one investigator calibrated by an experienced periodontist. A total of 20 patients (dental students) who were not part of this study was examined on two occasions separated by one week. Intra-examiner reliability was assessed using the kappa test. Kappa scores were 80% for PI, 82% for GI, and 80% for PPD. The results indicated good intra-examiner reliability. These subjects answered the questionnaire twice with a two-week interval. Reliability tests were carried out on all questions using Cronbach's alpha,^13^ which ranged from 0.85 to 0.92, indicating good internal consistency. 

### Statistical analysis

Data were entered into a personal computer and analyzed using the Statistical Package for Social Sciences (SPSS) software (SPSS^®^: Inc., Chicago, IL, USA). Frequency distributions were calculated for all the measured variables, and means were calculated for PI, GI, PPD and number of teeth with gingival recession. 

Chi-square and independent sample t-tests were used to detect differences in periodontal health knowledge and awareness among orthodontic patients, in respect to age, gender and duration of treatment. Backward stepwise linear regression analyses was used to determine the effects of the studied variables on periodontal health awareness among orthodontic patients. The level of significance was set at *p* ≤ 0.05.

## RESULTS

### Oral hygiene behavior

Ninety-four percent of orthodontic subjects (38% adolescents, 56% adults) reported they brush their teeth frequently. Sixty six percent (27% adolescents, 39% adults) brushed twice and 28% (11% adolescents, 17% adults) brushed three times daily. Of those, only 67% used auxiliary aids (interdental brushes and mouth washes). Six percent (2% adolescents, 4% adults) of orthodontic subjects admitted they do not brush at all. 

### Clinical examination

The means for PI, GI, PPD and number of teeth with GR for the studied subjects are shown in Table 1. Age, gender and duration of orthodontic treatment affected number of teeth with gingival recession, PI and PPD respectively (*p*< 0.05). 

**Table 1:** Means and standard error of the mean (SE) for PI, GI, PPD and teeth with GR among orthodontic patients according to age, gender and duration of treatment.

Variable	Age		Gender		Duration of treatment		Total
	Adolescents Mean (SE)	Adults Mean (SE)	Female Mean (SE)	Male Mean (SE)	<18 month Mean (SE)	≥18 month Mean (SE)	Mean (SD)
PI	0.75 (0.05)	0.69 (0.04)	0.67 (0.03)*	0.82 (0.06)*	0.70 (0.03)	0.79 (0.06)	0.72 (0.03)
GI	1.31 (0.04)	1.22 (0.04)	1.25 (0.04)	1.26 (0.05)	1.24 (0.03)	1.31 (0.07)	1.26 (0.03)
PPD	1.22 (0.29)	1.23 (0.03)	1.22 (0.02)	1.25 (0.04)	1.21 (0.02)*	1.30 (0.04)*	1.23 (0.02)
Teeth with GR	0.05 (0.22)*	0.07 (0.02)*	0.08 (0.03)	0.02 (0.02)	0.07 (0.03)	0.03 (0.02)	0.06 (0.02)

* P<0.05.

### Orthodontics patients’ knowledge of periodontal health (Table 2)

Seventy percent of the subjects identified what does bleeding gum indicate. However, 84% did not know what plaque is and 95% (94% females and 97% males; *p*= 0.015) did not know what does it cause. Only 24 subjects (8%) correctly answered all related questions. No significant differences were found among the studied groups. A high positive correlation was found between educational level of the subjects and their periodontal health knowledge (R^2^= 0.162, *p*= 0.005).

**Table 2:** Periodontal knowledge among orthodontic patients according to age, gender and duration of treatment (subjects with correct answers).

Questions	Age		Gender		Duration of treatment		Total n (%)
	Adolescents	Adults	Female	Male	<18 month	≥18 month	
	(n= 119) n (%)	(n=178) n (%)	(n=207) n (%)	(n=90) n (%)	(n=231) n (%)	(n=66) n (%)	
What is dental plaque?	29 (24%)	49 (27.5%)	48 (23%)	30 (33%)	60 (26 %)	18 (27%)	78 (26%)
	(X^2^= 0.655; P=0.884)		(X^2^= 3.346; P=0.341)		(X^2^=0.697; P=0.874)		
What can dental plaque cause?	3 (2.5)	12 (7%)	12 ( 6%)	3 (3%)	13 ( 6%)	2 ( 3%)	15 (5 %)
	(X^2^= 3.335; P=0.343)		(X^2^=10.506; P=0.015*)		(X^2^=0.811; P=0.847)		
What bleeding gum indicates?	79 (66%)	129 (72.5%)	152 (73%)	56 (62%)	161 (69 %)	47 (71%)	208 (70%)
	(X^2^=3.409; P=0.333		(X^2^= 4.334; P=0.228)		(X^2^=1.088; P=0.780)		
How can you prevent gum disease?	66 (55.5%)	102 (57%)	110 (53%)	58 (64 %)	130 (56%)	38 (58%)	168 (57%)
	(X^2^=3.409; P=0.333)		(X^2^= 5.120; P=0.163)		(X^2^=1.394; P=0.707)		

### Attitude of orthodontic patients toward fixed orthodontic treatment regarding periodontal health (Table 3) 

Negative attitude toward fixed orthodontic treatment in respect to periodontal health was observed in adult orthodontic patients (*p*< 0.001) and in subjects with orthodontic treatment duration of more than 18 months (*p*< 0.01). Gender differences were not detected. The questions more frequently answered negatively were: if regular dental visit contribute to enhance periodontal health (95%), if straight teeth are easier to clean (92%), if it is important to brush more after wearing fixed orthodontic appliance (87%) and if it is important to follow the advice and instructions in oral health given by clinician (79%). 

**Table 3:** Frequency distribution of orthodontic patients’ attitude toward fixed orthodontic treatment according to age, gender and duration of orthodontic treatment

Variable	Age		Gender		Duration of treatment		Total n (%)
	Adolescents (n= 119) n (%)	Adults (n=178) n (%)	Female (n=207) n (%)	Male (n=90) n (%)	<18 month (n=231) n (%)	≥18 month (n=66) n (%)	
Negative attitude (Scores 0-5)	52 (44%)	123 (69%)	124 (60%)	51 (57%)	128 (55%)	47 (71%)	175 (59%)
Positive attitude (Scores 6-9)	67 (56%)	55 (31%)	83 (40%)	39 (43%)	103 (45%)	19 (29%)	122 (41%)
(Chi square; P value)	(19.02; 0.000)***		(0.272; 0.346)		(5.295; 0.014)*		

*P<0.05, ***P<0.001.

### Orthodontic patients’ awareness of their periodontal health

The ajority of subjects were in the high level of awareness group (64%). Distribution of subjects according to age, gender and treatment duration is shown in Table 4. Age differences were detected (X^2^=6.97, *p*< 0.05). 

**Table 4:** Level of periodontal awareness among orthodontic subjects accords to age, gender and duration of orthodontic treatment.

	Age		Gender		Duration of treatment		Total
	Adolescents (n= 119) n (%)	Adults (n=178) n (%)	Female (n=207) n (%)	Male (n=90) n (%)	<18 month (n=231) n (%)	≥18 month (n=66) n (%)	
High awareness (scores 8-10)	86 (72%)	105 (59%)	132 (64 %)	59 (66%)	155 (67%)	36 (55 %)	191 (64%)
Moderate awareness (scores 5-7)	27 (23%)	51 (29%)	59 (29 %)	19 (21 %)	57 (25%)	21 (32 %)	78 (26%)
Low awareness (scores 1-4)	6 ( 5%)	22 (12%)	16 ( 8%)	12 (13 %)	19 ( 8%)	9 (13 %)	28 ( 9%)
Pearson Chi-Square	(6.97; P=0.031)*		(3.43; P=0.180)		(3.85; P=0.146)		

Frequency distribution for orthodontic subjects who were not aware of their periodontal health according to age, gender and duration of treatment is shown in Table 5. Age differences were found in respect to dental calculus (*p*< 0.001), dental stains (*p*< 0.001) and gingival recession (*p*< 0.001). Gender difference was detected in respect to dental plaque (*p*< 0.015). Duration of treatment affected awareness in respect to dental calculus (*p*< 0.01) and gingival enlargement (*p*< 0.001).

**Table 5:** Orthodontic subjects who were not aware of their periodontal health according to age, gender and duration of treatment.

Questions	Age		Gender		Duration of treatment		Total n (%)
	Adolescents (n= 119) n (%)	Adults (n=178) n (%)	Female (n=207) n (%)	Male (n=90) n (%)	<18 month (n=231) n (%)	≥18 month (n=66) n (%)	
Dental plaque	21 (18%)	36 (20%)	32 (16%)	25 (28%)	43 (19%)	14 (21%)	57 (19%)
	(X^2^=0.306; 0.346)		(X^2^=6.138; P=0.014*)		(X^2^=0.223; P=0.377)		
Dental calculus	17 (14%)	55 (31%)	49 (24%)	23 (26%)	47 (20%)	25 (38%)	72 (24%)
	(X^2^=10.72; P= 0.001***)		(X^2^=0.121; P=0.417)		(X^2^=8.59; P=0.004**)		
Dental stains	21 (18%)	65 (37%)	58 (28%)	28 (31%)	63 (27%)	23 (35%)	86 (29%)
	(X^2^=12.35; P=0.000***)		(X^2^=0.291; P=0.342)		(X^2^=1.432; P=0.149)		
Gingival bleeding	30 (25%)	59 (33%)	63 (30%)	26 (29%)	66 (29%)	23 (35%)	89 (30%)
	(X^2^=2.14; P=0.09)		(X^2^=0.071; P=0.451)		(X^2^=0.964; P=0.202)		
Gingival recession	10 (8%)	44 (25%)	40 (19%)	14 (16%)	37 (16%)	17 (26%)	54 (18%)
	(X^2^=12.76; P=0.000***)		(X^2^=0.599; P=0.274)		(X^2^=3.274; P=0.055)		
Halitosis	32 (27%)	49 (28%)	55 (27%)	26 (29%)	62 (27%)	19 (29%)	81 (27%)
	(X^2^= 0.015; P=0.506)		(X^2^=0.170; P=0.390)		(X^2^=0.098; P=0.433)		
Gingival enlargement	27 (23%)	49 (28%)	51 (25%)	25 (28%)	46 (20%)	30 (46%)	76 (26%)
	(X^2^=0.877; P=0.212)		(X^2^=0.325; P=0.333)		(X^2^=17.587; P=0.000***)		

*P<0.05, **P<0.01, ***P<0.001.

Orthodontic patients’ awareness toward their periodontal health during fixed orthodontic treatment demonstrated a significant association with attitude scores (R^2^= 0.132; *p*= 0.023), number of teeth with gingival recession (R^2^= -0.199; *p*=0.041), GI (R^2^= - 0.226; *p*=0.000), duration of treatment (R^2^= - 0.110; *p*= 0.047) and age (R^2^= - 0.160; *p*=0.006). 

## DISCUSSION

This study presented a comprehensive overview of the oral health behavior, knowledge, attitude and awareness of orthodontic patients toward their periodontal health. There was a 1:3 males to females’ ratio for the sample of treated patients. This finding reflects the fact that females are more concerned with their aesthetics, so they demonstrated better attendance to have their dentition maintained and checked, and thus were more represented in the sample. Sharma^14^ found that females seeking orthodontic treatment were approximately twice the males. 

Oral hygiene behavior of orthodontic subjects in this study was good. The majority of subjects reported brushing frequently, while only 6% admitted no brushing. This was expected since adequate oral hygiene level is requested before receiving any orthodontic treatment. Davies et al^15^ concluded that regular visits to the orthodontist are the most likely reason for improvement in oral hygiene and gingival health. However, Atassi and Awartani^16^ evaluated the oral hygiene status of patients with fixed orthodontic appliances and reported that 40% had fair oral hygiene and 60% had poor oral hygiene. The difference in the reported percentages may be due to variability of culture, availability of oral care services and different population. Oral hygiene behavior of orthodontic subjects in this study was similar to that reported by Baheti and Toshniwal.^5^

Despite the fact that the present study sample reported a good oral hygiene behavior, clinical examination revealed that they had developed generalized moderate gingivitis. This was in agreement with Zachrisson and Alnaes^17^ who demonstrated that, in spite of good cleaning with low plaque index scores, most children developed gen­eralized moderate hyperplastic gingi­vitis within one to two months after the placement of the appliances. However, other studies showed lower plaque and gingival index scores among patients with orthodontic treatment.^18^

Females had less plaque accumulation than males. This was in agreement with Kumar and Shristi,^19^ who reported that the females were more aware and had a better knowledge about dental health issues and more engaged in dental behavior than the male patients. However, the small sample size and the male/female ratio in this study makes this finding inconclusive.

Periodontal pocket depth was greater in subjects who used fixed appliance for more than 18 months. The plaque-retentive nature of orthodontic appliances increases plaque accumulation at the gingival margins, contributing to gingival inflammation and periodontal pockets. This was in agreement with Moosa et al,^20^ who showed that patients undergoing orthodontic treatment have increased plaque accumulation and probing depth, resulting in periodontal tissue destruction.

Teeth with gingival recession were more reported in adults group. This was in agreement with most studies^21^ ^,^ ^22^ that investigated gingival recessions and reported that periodontal tissue in younger patients has a more favorable response to orthodontic treatment than in older adolescents and adults. The pathogenesis of gingival recession may include brushing trauma; thin gingival tissue and underlying alveolar bone; and apical migration of the gingival margin, which location is determined by the axial inclination and alignment of the tooth. Joss-Vassalli et al^23^ suggested that treatment duration, age, gender or race did not have an influence on the development of recessions during treatment. 

Generally, public awareness of gum disease and particularly the role of dental plaque in relation to periodontal disease is poor, presumably due to inadequate health education concerning these conditions. Majority of orthodontic patients did not know what plaque is and what does it cause. This was in agreement with Azodo and Umoh,^24^ who reported that only 12.6% of the participants knew dental plaque as soft debris on teeth. Likewise, the majority of Jordanian adults^8^ who incorrectly defined the meaning of dental plaque, did not know the harmful effect of plaque and its role in the etiology of gingival disease. However, most of the study participants had a good level of knowledge regarding the role of oral hygiene in preventing gum disease, a finding that was reported in other studies.^8^ ^,^ ^25^

The majority of subjects in this study identified bleeding gum as a sign of periodontal disease. This was in agreement with Taani and Abu Alhaija,^26^ who suggested that gingival bleeding and enlargement were the two most common manifestations of periodontal disease that participants were aware of.

Most of orthodontic patients in this study had a high level of awareness of their periodontal health. They were aware of having dental calculus and dental stain, but not aware of having dental plaque. This may be due to the easy identification of stains and calculus on teeth. The identification of dental plaque is more difficult, since they do not know how it looks. Ajayi and Azodo^27^ assessed knowledge of oral health among Nigerian patients with fixed orthodontic appliances and reported that 93.5% of the subjects showed good oral health awareness. Baheti and Toshniwal^5^ showed that nearly 50% of the Indian patients were unaware about periodontal health.

In the present study, similar periodontal health awareness among boys and girls was recorded. This result was in contrast to the results of previous study,^28^ where females showed a higher oral health knowledge than males. 

In the present study, the majority of orthodontic patients had a negative attitude towards fixed orthodontic treatment in respect of periodontal health. This was in agreement with Baheti and Toshniwal,^5^ who reported that the attitude toward practice of oral hygiene among orthodontic patients was poor. However, most of them reported on the importance of oral hygiene measures and the need to follow these instructions. The negative attitude of orthodontic patients was increased by the longer duration of orthodontic treatment and age of the patients. This negative attitude may be caused by feeling tired and bored by the appliances, due to the increased duration of orthodontic treatment. 

Orthodontic patients’ awareness of their periodontal health during fixed orthodontic treatment demonstrated a significant association with their attitude, number of teeth with gingival recession, duration of treatment and age. This may be explained by the fact that subjects with negative attitude will not show interest to learn about periodontal health.

The results of this study indicated a poor knowledge, a moderate level of periodontal health awareness and a negative attitude among orthodontic subjects. This emphasizes the need to improve oral health education among orthodontic patients. Orthodontists through their long-term treatment procedure have opportunity and responsibility to educate their patients about periodontal health and to promote proper oral health behavior with emphasis on the prevention of periodontal disease. However, self-directed educational material such as a leaflet is an inexpensive and practical way of targeting large sections of the population to consider health change.^29^

Periodontal health knowledge among orthodontic patients, awareness of their periodontal health and their attitude toward periodontal health vary among different populations. Cultural differences, socio-economic status, educational background and availability of orthodontic services may explain these variations.^26^ ^,^ ^30^

Limitations of this study include small sample size with different female to male ratio, included subjects had different malocclusion with varying severities, and the subjects were recruited from a single orthodontic practice.

## CONCLUSION

» Periodontal health knowledge among orthodontic patients was poor.
» Periodontal health awareness among orthodontic patients was moderate.
» Orthodontic patients’ awareness of their periodontal health during fixed orthodontic treatment was affected by their attitude, number of teeth with gingival recession, duration of treatment and age.
» Orthodontic patients showed a negative attitude toward periodontal health. Patients negatively reported the need for regular dental visits during orthodontic treatment, the need for improvement of teeth brushing during fixed orthodontic treatment and the importance of following oral health advice.
